# Application of next generation sequencing in cardiology: current and future precision medicine implications

**DOI:** 10.3389/fcvm.2023.1202381

**Published:** 2023-06-23

**Authors:** Eirini Papadopoulou, Dimitra Bouzarelou, George Tsaousis, Athanasios Papathanasiou, Georgia Vogiatzi, Charalambos Vlachopoulos, Antigoni Miliou, Panagiota Papachristou, Efstathia Prappa, Georgios Servos, Konstantinos Ritsatos, Aristeidis Seretis, Alexandra Frogoudaki, George Nasioulas

**Affiliations:** ^1^ Genekor Medical S.A., Athens, Greece; ^2^ Third Department of Cardiology, Sotiria Hospital, Athens, Greece; ^3^ Unit of Inherited Cardiac Conditions and Sports Cardiology, First Department of Cardiology, National and Kapodistrian University of Athens, Athens, Greece; ^4^ Cardiology Department, “P. & A. Kyriakou” Children’s Hospital, Athens, Greece; ^5^ Second Department of Cardiology, Arrhythmia Unit, Evangelismos General Hospital of Athens, Athens, Greece; ^6^ Pediatric Cardiology Unit, “P. & A. Kyriakou” Children’s Hospital, Athens, Greece; ^7^ Unit of Inherited and Rare Cardiovascular Diseases, Onassis Cardiac Surgery Center, Athens, Greece; ^8^ Second Department of Cardiology, Attikon University Hospital, School of Medicine, National and Kapodistrian University of Athens, Athens, Greece

**Keywords:** next generation sequencing, personalized treatment, genetic analysis, cardiovascular diseases, cardiogenetics

## Abstract

Inherited cardiovascular diseases are highly heterogeneous conditions with multiple genetic loci involved. The application of advanced molecular tools, such as Next Generation Sequencing, has facilitated the genetic analysis of these disorders. Accurate analysis and variant identification are required to maximize the quality of the sequencing data. Therefore, the application of NGS for clinical purposes should be limited to laboratories with a high level of technological expertise and resources. In addition, appropriate gene selection and variant interpretation can result in the highest possible diagnostic yield. Implementation of genetics in cardiology is imperative for the accurate diagnosis, prognosis and management of several inherited disorders and could eventually lead to the realization of precision medicine in this field. However, genetic testing should also be accompanied by an appropriate genetic counseling procedure that clarifies the significance of the genetic analysis results for the proband and his family. In this regard, a multidisciplinary collaboration among physicians, geneticists, and bioinformaticians is imperative. In the present review, we address the current state of knowledge regarding genetic analysis strategies employed in the field of cardiogenetics. Variant interpretation and reporting guidelines are explored. Additionally, gene selection procedures are accessed, with a particular emphasis on information concerning gene-disease associations collected from international alliances such as the Gene Curation Coalition (GenCC). In this context, a novel approach to gene categorization is proposed. Moreover, a sub-analysis is conducted on the 1,502,769 variation records with submitted interpretations in the Clinical Variation (ClinVar) database, focusing on cardiology-related genes. Finally, the most recent information on genetic analysis's clinical utility is reviewed.

## Introduction

Cardiovascular diseases (CVDs) are a group of heterogeneous entities affecting the vasculature, the myocardium, and the electrical conduction system (1). They eventually lead to heart failure, which affects 1%–2% of the global population and is a major public health concern and leading cause of morbidity and mortality worldwide (2). Smoking, obesity, hypertension, and elevated cholesterol are among the risk factors for CVD; however, it is well known that only a small portion of disease cases are attributable to these traditional risk factors (3–5). In recent years, it has become evident that genetics plays a significant role in the emergence of many CVD conditions (1, 6). Relative to their genetic etiology, they can be subdivided into monogenic disorders, which are caused by the inheritance of one or two genetic variants and tend to cluster in families, and polygenic disorders, which involve multiple genetic variants and have a lower tendency to cluster in families (7). The main representative of a polygenic CVD is coronary artery disease (1, 8). Multifactorial CVDs are beyond the scope of the current review.

Monogenic disorders consist of both syndromic and non-syndromic abnormalities. They follow a Mendelian mode of inheritance and include cardiomyopathies, arrhythmic disorders, vascular disorders, and lipid disorders such as familial hypercholesterolemia. In accordance with the heterogeneity of signs and symptoms, they are also accompanied by large heterogeneity in the genetic etiologies responsible for the disease onset. The genetic features of inherited cardiovascular disorders are distinct. The disease is primarily caused by mutations in genes involved in appropriate cardiac function, and its inheritance pattern is typically dominant. Nevertheless, recessive X-linked inheritance has also been identified (9–11). In addition to missense, nonsense, and splicing variants, frameshift variants are also identified in the causative genes. Moreover, compound and digenic or oligogenic heterozygosity is reported in 5%–16% of the inherited cases (12, 13). Although uncommon, the presence of digenic alterations is associated with a more severe and complex phenotype observed in early-onset cardiomyopathies and channelopathies (14–18).

In the presence of a pathogenic variant, it is common to observe incomplete penetrance and variable expressivity of the disease, resulting in subclinical/silent disease, which complicates the detection of an inheritance pattern. In fact, pathogenic variants in genes associated with inherited CVDs are also present in the healthy population, as revealed by recent research that identified pathogenic variations in almost 1% (119/13.131) of asymptomatic elderly adults (mean age: 75 years) who were evaluated (19). Nevertheless, following the incomplete penetrance of such disorders, the number of presumed sudden cardiac deaths attributable to pathogenic or likely pathogenic variants detected in this population was relatively low (2/119 or 1.7% of carriers) and limited to genes associated with the LQT syndrome.

The inheritance pattern becomes more intricate in cases of *de novo* variants, which arise for the first time in a single family member, thereby constituting the first generation of carriers who can subsequently transmit this novel variant to their progeny. De novo mutations are primarily linked to syndromic cardiac disorders, although they have also been detected in diverse sporadic cardiomyopathies and arrhythmogenic diseases (20–23). Recent research indicates that *de novo* variants associated with cardiac and seizure disorders could account for up to 9% of sudden, unexplained pediatric deaths (24).

The genetic variability of cardiovascular diseases is shown by the variety of genes associated with a single CVD (19). For instance, the inheritance of Hypertrophic Cardiomyopathy (HCM) may be ascribed to more than 40 genes. Simultaneously, there is also phenotypic pleiotropy, resulting in the association of a single gene (i.e., *MYH7*) with several cardiomyopathies. Moreover, several types of cardiomyopathies and channelopathies have overlapping genes, which further complicates clinical diagnosis (25).

In the present review, we address the current state of knowledge regarding genetic analysis strategies employed in the field of cardiogenetics. Data and levels of evidence concerning inheritance and gene selection processes, as well as gene-disease association data compiled by multinational alliances such as the Gene Curation Coalition (GenCC), are reviewed. In addition, variant interpretation and reporting guidelines are discussed. An analysis of variants reported in the Clinical Variation (ClinVar) database is also included, to elucidate the utility of appropriate variant interpretation and reclassification based on the experience gained from the 136,116 variants with classification results available in cardiology-related genes (26). Finally, the most recent information on genetic analysis's clinical utility is assessed.

## NGS-based genetic diagnosis strategies

The increasing use of Next Generation Sequencing technology permits the analysis of multiple genes simultaneously at a low cost (27, 28). In addition, advances in computational and bioinformatics sciences enabled data management and interpretation of the results obtained. Therefore, the availability of such extensive genomic analysis platforms by a growing number of laboratories has allowed for a better comprehension of these disorders, while new genes and genetic alterations are constantly associated with an increased risk of monogenic CVDs (27). Moreover, for the same diagnosis, variations in diagnostic yield have been noted between studies, depending on the population investigated and the stringency of the criteria used for genetic testing (Table 1).

**Table 1:** NGS diagnostic yield in various cardiac disorders and number of genes implicated according to clinGen.

Disease	Prevalence	Diagnostic yield	Function of the main genes	No of Genes based on ClinGen	References
HCM	1:200	40%–72%	Sarcomere, nuclear envelope	28	(29)
DCM	1:2,500	8%–25% in sporadic DCM 30%–40% in familial DCM	Cytoskeleton, nuclear envelope, desmosome sarcomere (DCM + HCM + ARVC genes)	42	(30, 31)
ARVC	1:5,000	10%–50%	Desmosome, calcium homeostasis nuclear envelope, intermediate filament, Sarcomere genes,ion chanels	17	(32)
RCM	<1/10,00,000	10%–60%	Sarcomere cytoskeleton Z-disc (HCM+ DCM+ LVNC, +ACM genes)	-	(28, 32, 33)
LVNC	<1/10,00,000	10%–50%	Various (HCM+ DCM+ LVNC, +ACM genes)	-	(32)
LQT	1–2,500	50%–75%	Ion channels	10	(34–36)
SQT	<1/10,000 or unknown	20%–30%	Ion channels	4	(7, 37)
Brugada	1/2,000	20%	Ion channels	1	(7)
CPVT	1/20,000	50%–55%	Ion channels	7	(7, 38, 39)
TAAD (Syndromic and Non syndromic)	1/5,000–<1/1,000,000	25%–90%	Various	22	(40)
FH	1/300	30%–70%	Low-density lipoprotein receptor	4	(41–43)

HCM, Hypertrophic Cardiomyopathy; DCM, Dilated Cardiomyopathy; ARVC, Arrhythmogenic Right Ventricular Cardiomyopathy; RCM, Restrictive Cardiomyopathy; LVNC, Left Ventricular Non-Companion; LQT, Long QT; SQT, Short QT; CPVT, Catecholaminergic polymorphic ventricular tachycardia; FH, Familial Hypercholesterolemia; TAAD, Thoracic Aortic Aneurysms and Dissection.

In cardiology, multiple NGS-based approaches have been implemented. Some laboratories prefer to analyze panels of genes associated with a particular disorder due to the targeted nature of the analysis, which makes it simpler and more cost-effective. However, improvements in the NGS platforms' technology and the increase in their sequencing capacity permitted the accurate and fast analysis of a larger gene number simultaneously. Currently, several NGS approaches have focused on sequencing the coding regions and adjacent intronic regions of either the 5,000–7,000 clinically relevant genes (Clinically Exome Sequencing), or even of the about 20.000 genes that are known to be protein-producing (Whole Exome Sequencing, WES) (44).

Nevertheless, the optimal number of genes that should be included in NGS analysis has been a topic of investigation to avoid the inclusion of irrelevant and clinically insignificant genes. Understanding the genetic basis of various inherited cardiac conditions has aided in the selection of appropriate genes for inclusion in NGS panels. It is now well-established, for instance, that genetic analysis of Inherited Hypertrophic Cardiomyopathy (HCM) requires the investigation of sarcomeric genes, specifically, *MYBPC3, MYH7, MYL2, MYL3, TNNI3, TNNT2*, and *TPM1*. Variants in loci encoding non-sarcomeric proteins, such as desmosomal proteins or ion channels, have also been identified but account for a small proportion of HCM patients (29). Three additional genes, *PLN, FLNC*, and *ALPK3*, exhibit compelling evidence of causality (45, 46). Inherited HCM NGS panel analysis should also include gene alterations associated with HCM phenocopies, which are diseases whose phenotype resembles HCM but are caused by variants in distinct genes than typical HCM and account for 5%–10% of all HCM cases. The majority of these phenocopies are metabolic and lysosomal storage disorders (45).

In cases of familial Dilated Cardiomyopathy (DCM), NGS analysis has permitted the detection of pathogenic variants in numerous causative genes categorized based on the extent of cell damage they cause. The most prevalent gene categories associated with DCM are sarcomeric genes *(TTN, NYH7, ACTC1, TNNT2, TPM1*), the *LMNA* gene involved in nuclear envelope defects (laminopathies), genes encoding proteins comprising the cytoskeleton (such as *FLNA, DMD, DES*), and genes encoding desmosomes, which have also been reported in Arrhythmogenic Right Ventricular Cardiomyopathy (ARVC). Left ventricular dysfunction and the DCM phenotype have also been linked to other genes, such as *RBM20* (which regulates titin splicing) and *BAG3* (which encodes an antiapoptotic protein) (30, 31).

Arrhythmogenic Cardiomyopathy (ACM) is a broad and heterogeneous clinical entity that includes right and/or left ventricular involvement. ARVC, the best-characterized ACM, is regarded as a disease of the cardiac desmosome, with the *PKP2, DSP, DSC2, DSG2*, and *JUP* genes strongly associated with the disease (32). *PLN* is a non-desmosomal gene, that encodes phospholamban, a protein essential for calcium homeostasis, and has also been associated with ARVC pathogenesis, particularly in geographic regions with well-characterized founder variants (47). Variants in genes implicated in the nuclear envelope biochemistry, like *TMEM43, LMNA,* and *LEMD2* have also been correlated with ARVC. Additionally, alterations in the *DES* gene, which encodes the cardiac intermediate filament protein desmin, can result in right or biventricular forms of ACM. *DES* mutations are also associated with various other cardiomyopathies, including DCM, LVNC, and RCM (48). Likewise, sarcomere genes such as *MYH7, MYBPC3, MYL3, LDB3*, and *ACTN2* have been considered by several research groups as potential causes of ARVC (49, 50). Notably, alterations in cardiac electrophysiology-related genes such *RYR2, SCN5A*, and *PLN* have also been found in ACM patients (51).

RCM is a rare cardiomyopathy whose genetics are not fully understood. RCM-associated genes encode sarcomere, cytoskeleton, or Z-disc proteins. Remarkably, there is considerable genetic overlap with other cardiomyopathies, including HCM, DCM, LVNC, and ACM (33).

LVNC is also extremely rare in adults, but it is the third most common pediatric cardiomyopathy. The application of advanced NGS genetic testing has increased the number of genes associated with LVNC. These encode proteins with sarcomeric function, involved in cellular junction, role in signal transduction, cytoskeletal proteins, transcriptional/translational regulators, proteins with mitochondrial function, cellular junction proteins, and others (52).

Hereditary channelopathies of the cardiovascular system are a heterogeneous group of disorders. They include Brugada syndrome, Long QT syndrome (LQTS), Short QT syndrome (SQTS), and Catecholaminergic polymorphic ventricular tachycardia (CPVT). They are caused by alterations in genes involved in the ion channels, present in cell membranes and various organelles (53).

Heritable Thoracic Aortic Aneurysm and Dissection (HTAAD) is a collection of medical conditions that exhibit similar manifestations of aortopathy. These conditions may manifest with or without accompanying systemic features, which are respectively referred to as syndromic and nonsyndromic forms. The clinical presentations of patients with TAAD may vary due to distinct underlying etiologies and genetic defects (54). More than 50 genes with various degrees of evidence have been implicated in TAAD phenotypes (40).

The most prevalent inherited cardiovascular disease, familial hypercholesterolemia (FH) entails significant morbidity and mortality risks. FH is an autosomal dominant disorder most commonly associated with variants in the LDL receptor (*LDLR*) gene, although variants in the apolipoprotein B (*APOB*) gene, primarily in the LDLR-binding region of the protein, or gain-of-function variants in protein convertase subtilisin/kexin 9 (*PCSK9*) have also been detected. In addition, rare recessive FH is caused by variants in the LDLR adaptor protein 1 (*LDLRAP1*) locus.

Expert panels and cardiology societies are recommending the genes to be tested for each disease. More recently, the Clinical Genome Resource (ClinGen) cardiovascular clinical domain working group (CDWG) completed gene curation for inherited arrhythmia syndromes and cardiomyopathies (55–60, 59). Gene-Disease Validity Classifications have been completed for 165 genes. Of those 50 genes showed a definite correlation, while 81 genes exhibited moderate or limited correlation with such diseases. For 54 genes the correlation with the disease was either disputed or unknown. Considering only genes with definitive to limited evidence of disease association, the number of genes that should be evaluated in genetic testing based on the GlinGen gene curation is 28 for HCM, 42 for DCM, and 17 for ARVC (60, 61). Concerning the channelopathies, 20 genes were reported with limited to definitive association with Brugada syndrome, 10 with LQTS, 4 with SQTS, and 7 with CPVT (Table 1).

Guidelines currently recommend the focus analysis of genes with known correlation with the disease to avoid the detection of pathogenic variants in genes with uncertain association with the patient's phenotype (7, 62). Despite this, various laboratories use multiple gene panels, with some of the genes included having little or no evidence of disease association, which may provide insight into new disease-causing variants. For example, in HCM, 23 genes have testing recommendations, with 12 of them being definitively syndromic genes for which isolated left ventricular hypertrophy can be seen (62). However, due to expanded testing, new data concerning genes with previously limited data are also accumulating, increasing our knowledge of their impact on CVD heritability, such as the *ALPK3* gene recently defined by the Clingen effort as a gene with strong disease association, while more recently data concerning FHOD3 also suggest its role as a causal gene for HCM and eventually DCM (46, 63).

Therefore, a variety of scientific societies are aiming to give further evidence regarding the validity of gene-disease relationships. The information generated can be applied in the appropriate gene selection for the genetic analysis of cardiomyopathies or channelopathies and requires trustworthy and unified sources that describe the level of evidence for a gene's role in disease. Thus, to increase our knowledge about the gene-disease association, it is important to share data publicly and bring together groups involved in the assessment of the gene-disease validity process. This can be realized by international collaborations such as the Gene Curation Coalition (GenCC), with 12 reliable organizations participating including clinical testing providers, expert panels, and committees with curated knowledge bases (64). The aim is to create uniform terminology for gene curation activities, facilitating the consistent assessment of genes that have been reported to be associated with diseases. The GenCC database contains classifications for 4,629 Genes (accessed in May 2023). Participants report, for each gene, the observed association with a particular disease, using standardized criteria for varying levels of gene-disease clinical validity. From greatest to lowest degree of evidence, the following terms are used to describe gene-disease associations: definite, strong, moderate, supportive, limited, disputed, animal model only refuted, and no known disease relationship). Considering that each participant renders his own assessment of the gene association to a particular trait, diverse outcomes might be noticed among participants. Based on this observation and considering that the collective consideration of all participant conclusions could be more informative, we have categorized each gene in relation to a phenotype by considering the verdicts of all participants. Therefore, a numeric value was assigned from 8 to −2 based on the strength of the evidence. 8 for the definitive gene-disease association, 6 for strong, 5 for moderate, 4 for supportive, 3 for limited 2 for disputed, 1 for animal model, and −1/−2 for refuted, no known disease relationship. Then, using the mean value (consensus Level of Evidence, mLoE) acquired by various submitters for each gene, we generated a classification system in which any value >1 may be regarded as significant and the gene in consideration is thus eligible for inclusion in panel analysis for the associated disease. Among the 178 cardiovascular disease-related genes described, 150 demonstrated a significant degree of correlation with the condition (>1) and should therefore be included in genetic analysis.

**Figure 1:**
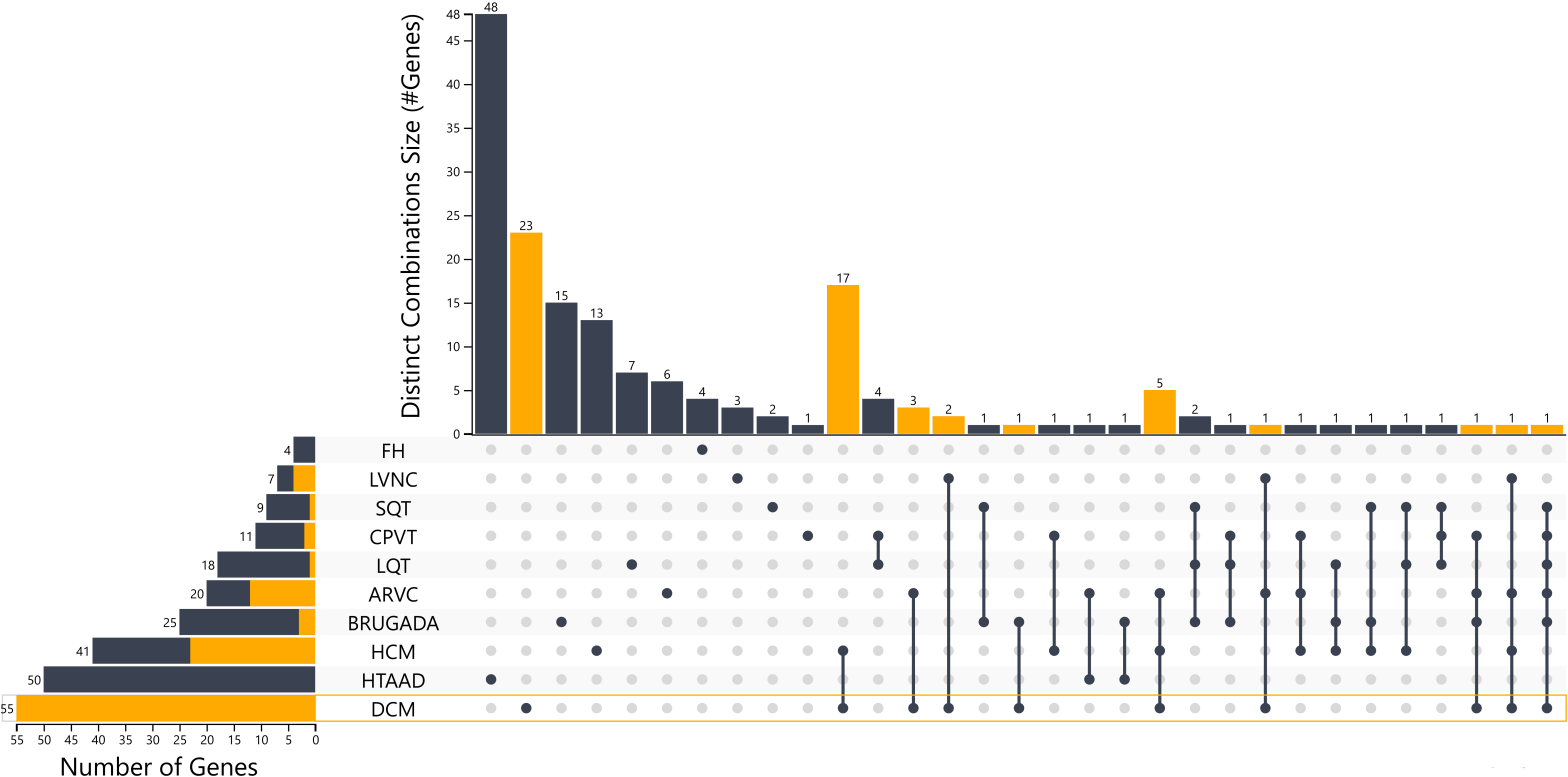
Upset plot of the different gene sets among the cardiovascular disease-related genes with the distinct combinations among categories. An interactive version of the plot is also in the supplementary files section and in the link https://tinyurl.com/upsetplot. DCM is highlighted as an example where 23/55 of the associated genes are unique for DCM and the remaining 32 show overlap among the different groups as shown in orange and through the lines that connect the dots at the level of DCM.

Using this approach, 41 HCM, 55 DCM, 20 ARVC and 7 LVNC genes should be included in gene panel analysis for cardiomyopathies. Moreover, for LQTS, SQTS, CPVT and Brugada syndrome, 18, 9, 11 and 25 genes respectively have a positive correlation with these syndromes. Of note the more submissions are added, the more accurate the numeric gene-disease association would be. An upset plot of the different gene sets among the cardiovascular disease-related genes is shown in Figure 1. Table 2 displays the genes associated with inherited cardiological conditions in the GenCC database, as well as their consensus Level of Evidence. It should be emphasized, however, that due to the heterogeneity of cardiac conditions and the overlapping phenotypes, a focused analysis on a specific cardiomyopathy or arrhythmogenic syndrome may overlook the genetic etiology of the disease. For example, RCM-related gene submissions are absent, possibly due to the low prevalence of this condition. Moreover, genes such as *FLNC, DES*, and *CRYAB* are known to be associated with RCM, they are not reported in the GenCC database or are associated with other cardiomyopathies (33). Similarly, *ILK* and *LEMD2* gene mutations are associated with ARVC, whereas *MIPEP* gene alterations are detected in LVNC but not reported in the database (11, 65, 66). Consequently, regardless of the suspected cardiomyopathy type, it is advised to perform a comprehensive analysis for cardiomyopathies.

**Table 2:** Genes with various levels of evidence based on the consensus classification of submissions in the GenCC database.

Disease	Definitive/strong genes (cLoE > 5)	Moderate genes (5 ≥ cLoE > 4)	Supportive Genes (4 ≥ cLoE > 3)	Limited genes limited (3 ≥ cLoE > 2)	Disputed genes (2 ≥ cLoE ≥ 1)
HCM	*ACTC1, ALPK3, COQ9, CSRP3, FLNC, MRPL44, MTO1, MYBPC3, MYH7, MYL2, MYL3, PRKAG2, TNNI3, TNNT2, TPM1*	*FHOD3, JPH2, PLN, TNNC1*	*TTN*	*ANKRD1, CALR3, CAV3, KLF10, MYH6, MYLK2, MYOM1, MYOZ2, MYPN, NEXN, OBSCN, PDLIM3, RYR2, SMYD1, TCAP, TRIM63, VCL*	* CACNB2, CASQ2, DSP, KCNQ1*
DCM	*BAG3, DES, DMD, DSP, FLNC, LMNA, MYH7, PLN, RBM20, SCN5A, TTN, TNNC1, TNNT2*	*ACTC1, ACTN2, JPH2, NEXN, PPCS, TNNI3, TPM1*	*ABCC9, LAMA4, VCL*	*ANKRD1, CSRP3, CTF1, DSG2, DTNA, EYA4, FKTN, GATAD1, ILK, LDB3, MYBPC3, MYBPHL, MYH6, MYL2, MYPN, NEBL, NKX2-5, OBSCN, PLEKHM2, PRDM16, PSEN2, RAF1, RPL3l, SGCD, TBX20, TCAP, TNNI3K, TXNRD2*	*MYL3, PDLIM3, PKP2, PSEN1*
ARVC	*DSC2, DSG2, DSP, JUP, PKP2, TMEM43*	*DES, PLN*	*CDH2*	*CTNNA3, LMNA, MYBPC3, MYH7, MYL3, RYR2, SCN5A, TGFB3, TJP1, TTN*	*LDB3*
RCM	*–*	*–*	*–*	*–*	*–*
LVNC	*MYBPC3, MIPEP*	* *	*PRDM16*	*BMP10, DSG2, DTNA, SYNE2*	* *
LQT	*CALM1, CCALM2, CALM3, KCNE1, KCNH2, KCNQ1, SCN5A, TRDN*	*CACNA1C,*	*CAV3*	*KCNJ2, KCNJ5, RNF207, SCN4B, SNTA1*	*AKAP9, ANK2, KCNE2, KCNJ5, SCN4B, SNTA1*
SQT	*KCNH2, KCNQ1*	*KCNJ2, SLC4A3*	* *	*CACNA1C, CACNA2D1, CACNB2, SCN5A, SLC22A5*	* *
Brugada	*SCN5A*	*SCN1B*	* *	*ABCC9, CAV3, DEPDC5, DLG1, HCN4, KCNH2, RANGRF, PRAD*	*ANK2, CACNA1C, CACNA2D1, CACNB2, GPD1l, KCND3, KCNE3, KCNE5, KCNJ8, PKP2, SCN10A, SCN2B, SCN3B, SLMAP, TRPM4*
CPVT	*CALM1, CALM2, CASQ2, RYR2, TECRL, TRDN*	*CALM3*	* *	* *	*ANK2, KCNJ2, PKP2, SCN5A*
Syndromic and Non syndromic familial TAAD	*ACTA2, ADAMTS2, AEBP1, ALDH18A1, ATP6V0A2, ATP6V1E1, B3GALT6, B4GALT7, C1R, CHST14, COL1A1, COL1A2, COL3A1, COL5A1, COL5A2, DSE, EFEMP2, ELN, FBLN5, FBN1, FKBP14, LOX, LTBP4, MYH11, MYLK, PLOD1, PRKG1, PYCR1, SLC39A13, SMAD2, SMAD3, TGFB2, TGFBR1, TGFBR2, TNXB*	*ATP6V1A, BGN, C1S, FOXE3, MFAP*	*THSD4*	*ADAMTSL2, ATP6V0D2, FBN2, FLNA, HCN4, MAT2A, NOTCH1, SLC2A10, TGFB3*	* *
FH	*APOB, LDLR, LDLRAP1, PCSK9*	* *	* *	* *	* *

HCM, Hypertrophic Cardiomyopathy; DCM, Dilated Cardiomyopathy; ARVCM, Arrhythmogenic Right Ventricular Cardiomyopathy; RCM, Restrictive Cardiomyopathy; LVNC, Left Ventricular Non-Companion; LQT, Long QT; SQT, Short QT; TAAD, thoracic aortic aneurysm and aortic dissection; CPVT, Catecholaminergic polymorphic ventricular tachycardia; FH, Familial Hypercholesterolemia; TAAD, Thoracic Aortic Aneurysms and Dissection.

Obtaining high accuracy and sensitivity of NGS genomic analysis is of great importance. Hence, validated NGS methodologies should be used, capable to detect all types of genetic variations, videlicet, Single Nucleotide Variations, small insertions, and deletions as well as intragenic Copy Number Variations (CNVs). For a long, the platform of choice to detect genome-wide CNVs has traditionally been Chromosomal Microarrays (CMA), while multiplex ligation-dependent probe amplification (MLPA) was applied for the detection of smaller intergenic CNVs. However, due to bioinformatics and methodological improvements, those platforms tend to be replaced by highly sensitive NGS technologies. NGS analysis of intragenic CNVs can increase the diagnostic yield in several cardiac disorders since they represent a minor but important percentage of the genomic variations observed. A recent NGS study for example indicated that such alterations are presently detected in about 7% of Arrhythmogenic cardiomyopathy cases negative, for pathogenic point variants in desmosomal genes (67). A different study detected CNVs in 2 percent of the individuals with sudden cardiac death (68). While in Congenital heart diseases the contribution of CNVs seems to be between 10% and 15%, the combined use of NGS offers the greatest diagnostic accuracy (69).

Even though analysis of genes included in guidelines can provide meaningful information and guide clinical management of patients without unnecessary confusing information, there is also the concern that guidelines-guided genetic analysis could miss genetic information. On the other hand, it should be noted that the technology today offers the possibility of large panels or even WES and WGS analysis with minimum effort and at no additional cost. Such a genetic analysis strategy generates results that could be used for diagnosis and patient management using the information currently available, but it can also enrich our knowledge on the genetic basis of the disease and provide the basis for new gene-disease associations. Thus, genetic results with apparent low clinical utility today could become significant in the future as the information from large NGS panels is accumulating, without the need to repeat the analysis. Moreover, a recent study including almost 4,800 patients with suspicion of cardiomyopathy or arrhythmias analyzed by NGS, has shown that a comprehensive NGS analysis approach could identify cases that would have been missed due to misdiagnosis (70). Based on these results, 10.9% of the variant-positive cases would have been missed. In the majority of cases, the gene alteration detected indicated a different diagnosis but within the same broad category of the diagnostic indication (e.g., genetic testing identified different cardiomyopathy than that indicated by the referral). Notably though, 27 patients (36.0% of the misdiagnosis group; 3.9% of all 689 patients) had an arrhythmia diagnosis but a positive test result for cardiomyopathy or vice versa. Another important result from this study is that the use of strict diagnostic criteria for test referral could obstacle to genetic diagnosis. In fact, 137 positive results (14.4%) would have been missed, if only individuals with a high suspicion of hereditary cardiomyopathy or arrhythmia had been evaluated (70).

However, a broad genetic test is often challenging in terms of data analysis. Hence, it has been shown that even though the diagnostic yield in genetically heterogeneous disorders can be increased by using bigger gene panels in clinical exome and genome analysis, they often prioritize variants with likely benign effects. Thus, such an approach requires extreme caution in the analysis and prioritization of candidate variants, as well as the careful phenotypic selection of the individuals to undergo panel testing (71). Furthermore, inevitably the analysis of more genes also increases the number of variants with an uncertain significance which is an additional source of confusion.

The utilization of WES/WGS techniques allows for a comprehensive analysis or selective examination of genes associated with a patient's phenotype. This can be achieved through the appropriate selection of genes and the development of virtual NGS panels, relevant to the attributed phenotype. This approach facilitates the analysis of a greater number of genes associated with a particular phenotype, while the genetic data acquired can be easily retrieved in the event of the discovery of a novel gene linked to the patient's ailment or the emergence of an alternative disorder in the subject under examination. The additional information might also be useful in the research context, that is, improving our knowledge of gene-disease relationships. Furthermore, the comprehensive nature of the analysis provides the opportunity to apply a more expanded evaluation, in case of initially negative results, considering the possibility of other genetic disorders with similar phenotypic manifestations.

### Variant interpretation

Classification and interpretation of variants are essential components of NGS analysis. Numerous genes are examined, the number of data that need categorization based on their pathogenicity rises exponentially, making analysis challenging. For instance, it is anticipated that the analysis of 100 genes would produce a median of over 250 variations compared to a reference genome, while a single gene analysis would produce less than 2–3 of such variants (72). Nonetheless, only a few of these variations have a frequency in the general population of less than 1%, and among these the eventual presence of a gene alteration with a definitive association with the disease should be identified. The complexity of the analysis increases exponentially in the case of WES and even more in WGS. Therefore, determining their clinical importance is difficult and needs sophisticated bioinformatics methods for accurate categorization (73). In 2015, the American College of Medical Genetics and Genomics (ACMG) and the Association for Molecular Pathology (AMP) published standards and guidelines for the interpretation of sequence variants (74). They provided criteria and levels of evidence for the classification of the variants as “pathogenic” (P), “likely pathogenic” (LP), “uncertain significance” (VUS), “likely benign” (LB) or “benign” (B). Following the release of the guidelines, both general and disease-specific criteria have been developed to facilitate the precise implementation of ACMG/AMP evidence types (59, 75).

As previously mentioned, a substantial proportion of variations detected in cardiology genetics are missense variants. This further complicates the categorization of variants, since their classification is more difficult than that of an almost invariably pathogenic stop codon or a splice site alteration.

In addition, before the period of huge public databases, if an amino acid change was found in a patient with a monogenic heart disease, its frequency was compared with a small number of persons in the control group, providing the false impression that it was disease-specific. In recent years, however, information on genetic variation has been accumulated in hundreds of sequenced control exomes, allowing for a more accurate calculation of the frequency of variations in the general population. In addition, the underrepresentation of individuals from varied racial and ethnic origins in genome wide association studies (GWAS) led to the misidentification of benign variations as pathogenic, particularly in non-European groups (76, 77).

According to research, a considerable number of claimed disease-causing variations were also detected in ExAC, albeit at a frequency incompatible with cardiomyopathy causation (6.5% of HCM, 11.9% of DCM, and 13.5% of ARVC variants are present at MAF > 104). ExAC refinement enables the exclusion of 75% of HGMD variants that could not be eliminated as disease-causing using the Exome Sequencing Project (the biggest control data set prior to ExAC) owing to a single allele count (76).

In addition, the advent of ACMG guidelines on variant classification has provided the appropriate rules for a uniform, unambiguous, and accurate classification across labs. Prior to then, labs used varying standards for classifying a variation as pathogenic or likely pathogenic. As a result of this occurrence, a number of benign or likely benign variants were erroneously categorized as pathogenic, resulting in misdiagnosis and consequential implications for patient and family management (78–80).

Currently, the Genome Aggregation Database (gnomAD) is utilized as a tool for aiding in variant classification. This resource comprises 125,748 exome sequences and 15,708 whole-genome sequences obtained from unrelated individuals who were sequenced as part of diverse disease-specific and population genetic studies (81).

The implementation of a threshold for allele frequency is advised in order to differentiate between rare and common variants. When an atypical variant is detected and considered to be a possible indication of a genetic heart condition, it should be either absent or very rare in the gnomAD database. Regarding HCM, the pathogenic criterion PM2 is activated for variants that exhibit a frequency of less than 0.004% in population databases, which suggests their scarcity or non-existence (59, 75). This standard is enforced with greater stringency for conditions that have lower incidence rates. Furthermore, the determination of a variant's pathogenicity is based on a range of ACMG criteria that consider multiple factors, including the variant's type and location, computational and functional data, as well as segregation data.

Functional analyses *in vivo* and *in vitro* could lead to a better understanding of the variant's impact on the protein's function. This has been shown in patients with cardiac conduction system disease, where 7/21 VUS were reclassified to LP based on the cellular electrophysiological study and *in vivo* zebrafish cardiac assay (82). Moreover, *in vitro* analysis of *DES* missense variants showed that the N-terminal part of the 1A coil domain is a hot spot for pathogenic variants, affecting desmin filament assembly (83).

Segregation data can also support either a benign or a pathogenic classification. The presence of a phenotype or disease in multiple affected family members or in individuals across multiple families associated with a particular variant provides supportive evidence for pathogenicity (PP1). On the contrary, the absence of segregation between a variant and a disease can serve as proof to endorse a benign classification (BS4) (73). Relying solely on cosegregation data cannot be considered conclusive evidence for pathogenicity, regardless of the strength of the evidence supporting it. This is due to the possibility that the variant in question may be in linkage disequilibrium with the actual pathogenic variant. Furthermore, the potential for incomplete penetrance and variable expressivity of the disease may present a challenge in interpreting these findings (74).

### Variant reclassification and ClinVar data analysis

The Clinical Variant database (ClinVar) integrates knowledge concerning genetic variation and its association with human disease. Examining these data may provide insight into the genes implicated in various diseases and the detected variant type. Until August 2022, there are 1,502,769 variation records with submitted interpretations (Unique variation records with interpretations) specific to a gene (13,236 genes). Of these variation records, 136,708 have been classified as pathogenic (P), 55,202 as Likely Pathogenic (LP), 590,985 as VUS, 411,555 as Likely Benign (LB) and 227,282 as Benign (B). While for 65,505 variants there are Conflicting interpretations. These are the outcomes of distinct classifications of a variant by different laboratories. They include submissions conducted prior to the implementation of the ACMG guidelines or incorrect use of the guidelines by some laboratories for variant categorization. In certain cases, information unavailable to other submitters, such as segregation or phenotypic data, could result in the classification of a variant to a higher or lower category (for example, LP or LB instead of VUS) compared to other submitters.

Among ClinVar submissions, 136,116 variants occur in the 143 genes associated with hereditary cardiac disorders reported in GenCC. The majority of these variants (82,324/136,116, 60.48%) pertain to 42 genes with a definitive correlation with CVD disorders, reflecting the fact that these gene categories are more thoroughly studied than others. Concerning the variant type in CVDs, 59,203 were missense and 13,164 were PVS1 which are defined as “null variant (nonsense, frameshift, canonical ±1 or 2 splice sites, initiation codon, single or multi-exon deletion) in a gene where loss-of-function (LoF) is a known mechanism of disease” (84).

The classification rates for all cardiology-associated genes in ClinVar were as follows: 10,442 (7.67%) P, 5,676 (4.17)% LP, 58,178 (42.74%) VUS, 37,967 (27.89%) LB, 13,125 (9.64%) B and 9,862 (7.25%) with conflicting interpretation. In addition, the P/LP classification rate for definitive/strong genes was significantly higher than for other gene categories (16.10% vs. 3.82%, P0.0001), indicating their substantial contribution to hereditary CVDs (Table 3).

**Table 3:** Percentage of variants’ classification in correlation to gene category.

Gene category	Number of Genes	% P/LP	% VUS	% Conflicting
DEFINITIVE_STRONG genes	55	16.10%	40.90%	8.57%
MOD_SUP_LIM_OTHER genes	88	3.82%	47.20%	4.88%
All genes	143	11.92%	43.02%	7.29%

**Figure 2:**
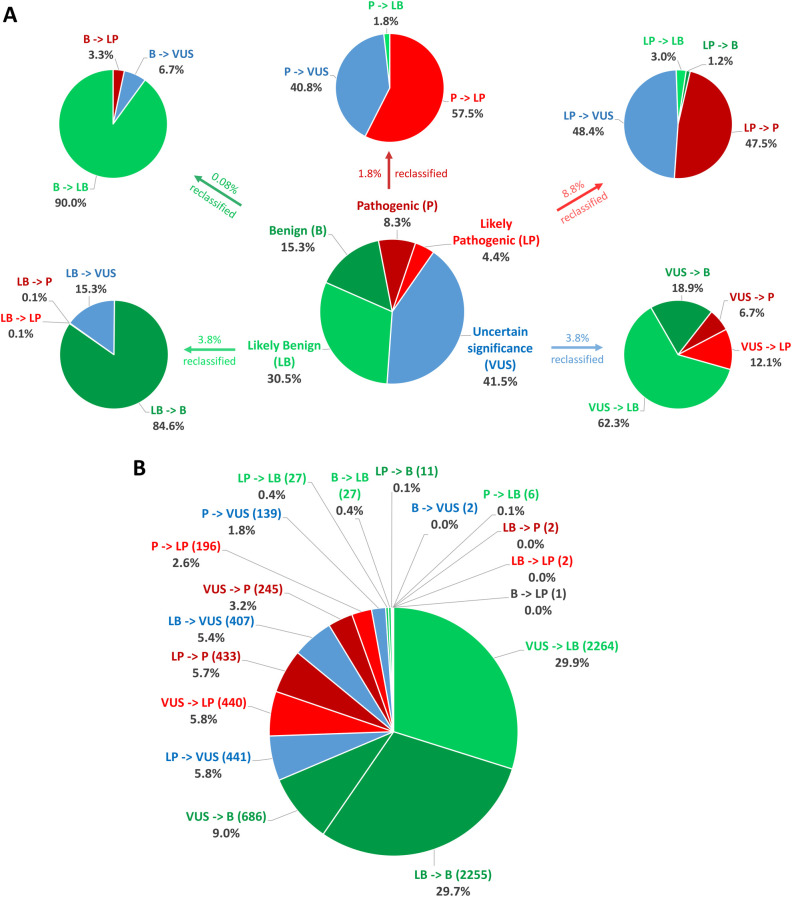
Statistics on classification and reclassification from ClinVar (Aug 2016–Aug 2022) for genes related to cardiac diseases. (**A**) The distribution of the 232,025 starting classifications for genes related to cardiac diseases to the five standard ACMG/AMP classification terms and reclassification statistics for each category relative to the percentage of the initial classification group. (**B**) Percentages relative to the number of all reclassifications.

In total, between 2016 and 2022, 232,025 classifications were submitted to ClinVar using one of the five standard ACMG/AMP classification terms, for genes related to cardiac diseases. By August 2022, only 3.27% (7,584/232,025) of these categories had been reclassified and updated in ClinVar by the submitter. Among these reclassifications, 20.56% (1,559/7,584) were moved to a higher classification category (VUS to LP/P, LP to P, LB/B to VUS, LB/B to LP/P), while 79.44% were downscaled. Of the five classification terms, 3.78% (3,635/96,231) of the variants initially classified as VUS were reclassified, with 18.85% (685/3,635) of the reclassification being upgraded to the P/LP category and 81.16% being downscaled as B/LB (Table 4, Figure 2).

**Table 4:** Summary of classification and reclassification from ClinVar (Aug 2016–Aug 2022) for genes related to cardiac diseases (adapted from (163).

Starting classification (*n*)	Percentage reclassified (*n*)	Reclassification type (*n*)	Percentage of the initial classification group	Percentage of all reclassifications
Pathogenic (19,204)	1.78% (341)	P → LP (196)	57.5%	2.6%
		P → VUS (139)	40.8%	1.8%
		P → LB (6)	1.8%	0.08%
		P → B (0)	0.0%	0.00%
Likely pathogenic (10,319)	8.84% (912)	LP → P (433)	47.5%	5.7%
		LP → VUS (441)	48.4%	5.8%
		LP → LB (27)	3.0%	0.4%
		LP → B (11)	1.2%	0.2%
Uncertain significance (96,231)	3.78% (3,635)	VUS → P (245)	6.7%	3.2%
		VUS → LP (440)	12.1%	5.8%
		VUS → LB (2,264)	62.3%	29.9%
		VUS → B (686)	18.9%	9.1%
Likely benign (70,683)	4.50% (2,666)	LB → P (2)	0.08%	0.03%
		LB → LP (2)	0.08%	0.03%
		LB → VUS (407)	15.3%	5.4%
		LB → B (2,255)	84.6%	29.7%
Benign (35,588)	0.08% (30)	B → P (0)	0.0%	0.00%
		B → LP (1)	3.3%	0.01%
		B → VUS (2)	6.7%	0.03%
		B → LB (27)	90.0%	0.4%

B, Benign; LB, likely benign; LP, likely pathogenic; P, pathogenic; VUS, variant of uncertain significance.

Variants in genes having a definite association with cardiovascular disease are more likely to be upgraded in the reclassification, with 23.45% of the resubmissions upgrading them, compared to 5% to 15% in the other gene subclasses (strong to unknown effect genes). This may be a result of the more precise classification of such frequently mutated and, thus, well-studied genes. In addition, it may be deduced that the strongest correlation of genes with cardiac conditions frequently leads to variants in them being confirmed as disease-causing. The top 5 genes with most reclassifications (>300) are *FBN1, TTN*, *MYH7, SNC5A* and *RYR2*. Among these, *FBN1* and *MYH7* have a large proportion of upgraded VUS (48.21% and 70.62%, respectively). Both are genes classified as definitive for cardiac disorders with a significant number of missense variants. Such variants are more challenging to classify than others, such as frameshift variants, thus it is sensible to require regular reclassification, which will upgrade them more frequently in genes closely associated with the disease (and thus more probable to carry variants with pathogenic effect). In fact, 75.27% of the VUS in ClinVar are missense variations, reflecting the difficulty in classifying these alterations, as compared to 2.84% of VUS variants belonging to the PVS1 variant category.

**Figure 3:**
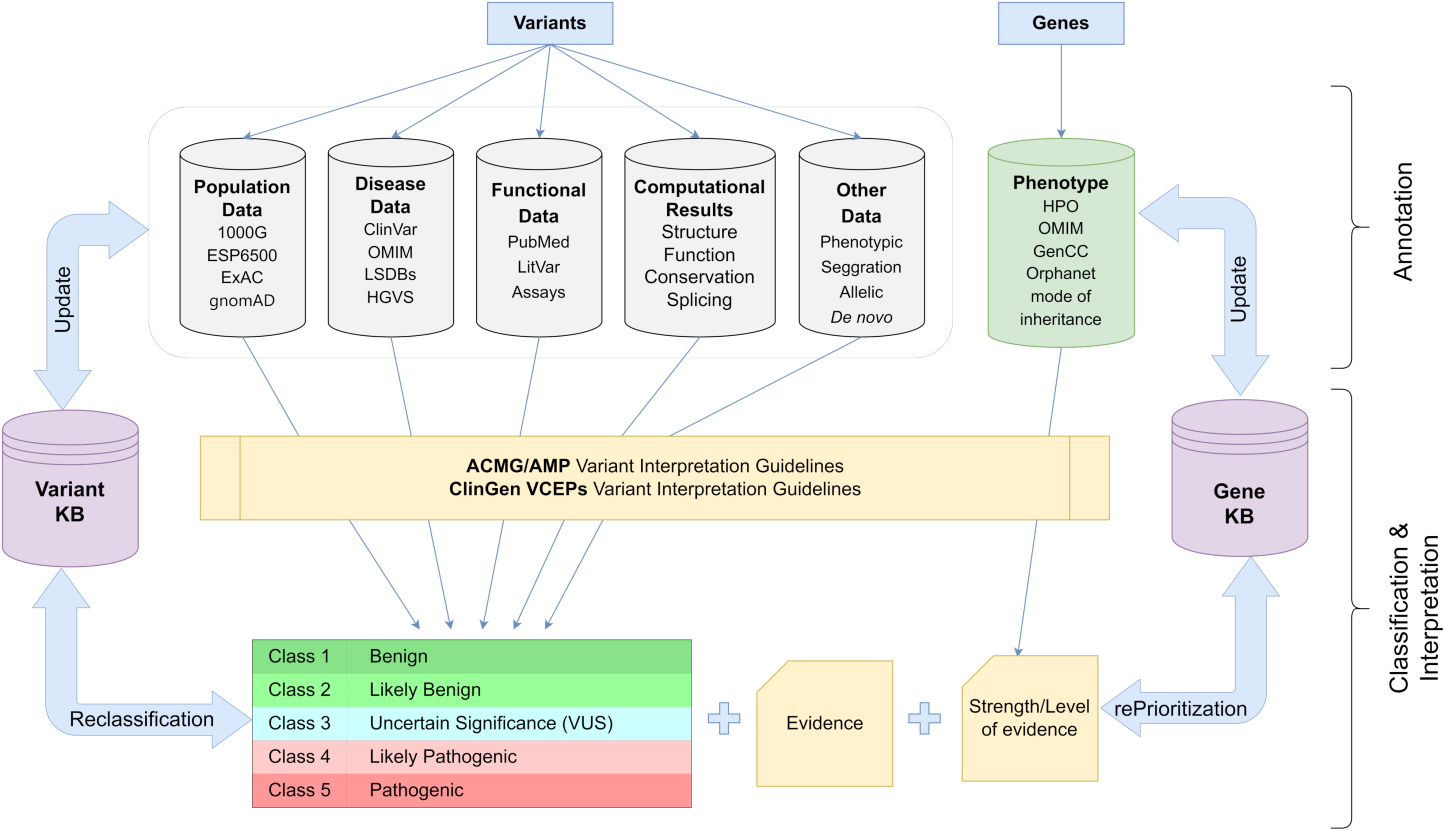
Schematic representation of the workflow used for appropriate classification and interpretation of the genetic variants in clinical practice (details in text). 1000G, 1,000 Genomes; ESP6500, NHLBI Exome Sequencing Project; ExAC, Exome Aggregation Consortium; gnomAD, Genome Aggregation Database; ClinVar, Clinical Variation database; OMIM, Online Mendelian Inheritance in Man; LSDBs, Locus Specific Databases; HGVS, Human Genome Variation Society; HPO, Human Phenotype Ontology; ACMG, American College of Medical Genetics and Genomics; AMP, Association for Molecular Pathology; ClinGen, Clinical Genome Resource; VCEP, Variant Curation Expert Panel.

Therefore, the determination of the pathogenicity of each alteration must be based on publicly accessible data from demographic and disease databases as well as published functional information. In addition to phenotypic and segregation analysis results, the annotation procedure should also take into consideration computational data on the influence of the variant on protein function. Based on the existing criteria, the variation should then be categorized into one of five pathogenicity classifications. In addition, it is suggested that each finding is categorized according to a disease and inheritance pattern. In cardiology genetics, careful consideration should also be given not only to the identified variation but also to the disease-causing genes. Pathogenic results in genes with insufficient evidence of association to the patient's phenotype should be eliminated from the analysis or explicitly distinguished from variations with strong/moderate disease association. This procedure should be dynamic, allowing for reevaluation of the variant's pathogenicity if new information becomes available in the future (76, 85, 86) (Figure 3).

A major constraint of NGS analysis in which numerous genes or exomes are sequenced concurrently is that it generates a large number of variations that cannot be definitively classified as pathogenic or benign. According to the recommendations, these variations are classified as variants of uncertain significance (VUS) and should not be used in clinical decision-making (32, 74). The frequency of VUS discovered varies across studies and is dependent on the number of genes tested and the stringency of the categorization criteria. A VUS may be reclassified in the future if information that was unavailable at the time of the initial classification becomes available. This may be accomplished through segregation analysis to see whether the variant segregates with the disease in other family members of the proband, or RNA analysis to determine the influence of a splicing variant on the splicing process. In addition, the expanding use of NGS technology facilitates the gathering of further knowledge regarding genes and variations, hence facilitating the future reclassification of VUS (87).

Even though the detection of VUS has no effect on the management of the disease, they should be included in clinical reports as they have the potential to be reclassified as pathogenic or likely pathogenic (P/LP) or benign or likely benign (B/LB) in the future. In the categorization and reporting of VUS, all publicly accessible information concerning variant carriers' phenotype, data about the segregation of the variation in families, and any existing functional analysis data should be examined. In addition, labs should be able to maintain a registry of identified variations and reexamine and reclassify them regularly when new information becomes available (87). In addition, whenever a VUS reclassification occurs, the affected individual must be promptly notified. In order to expedite VUS reclassification, the exchange of data across labs conducting such tests, which might increase the available knowledge on the impact of a variant, would be of tremendous use. Thus, it is essential to register any variations identified in publicly accessible databases such as Clinvar, a repository where the relationship between medically significant variants and phenotypes is documented (26).

Recent studies have shown, however, that the majority of VUS are eventually reduced to benign or probable benign (78–80). Therefore, clinicians should be extremely cautious with VUS management, as the erroneous use of such variants as pathogenic could have detrimental effects not only for the proband but also for his relatives, who could receive false information regarding their likelihood of disease inheritance and whose clinical management could be incorrectly altered through cascade testing.

In cardiology, apart from the VUS there is also an additional problem, which is the genes without validated association with a cardiac condition, often indicated as GUS (Genes of Uncertain Significance) (74). In case such genes are included in an NGS analysis they should be explicitly differentiated from those with strong/moderate evidence of disease association. Thus, their limited evidence should be highlighted and as for the VUS, clinicians, and patients should be informed in case such genes are down or upgraded in the future (7). The number of such genes and their impact on disease onset is expected to be elucidated in the future, with the augmentation of the genetic tests performed and the assistance of expert panels such as ClinGen.

In addition, several ethical considerations may arise whenever a genetic test is conducted. Before conducting clinical NGS, healthcare providers should offer genetic counseling services and secure informed consent from the individual tested. It is imperative to securely preserve the data produced to prevent the disclosure of sensitive information about the genetic profile of the subject under examination. Furthermore, utilizing this data for research purposes should only be done with explicit consent. Also, the laboratories should not proceed with the re-analysis and reporting of novel findings, unless explicitly requested to do so or for the purpose of ensuring quality assurance. In addition, the implementation of WES or WGS could lead to the identification of an immense number of findings, the vast majority of which lack established correlation or are not pertinent to the individual's medical condition. Consequently, the outcomes of such a test, particularly an analysis of the entire genome, may not be conclusive but only generate hypotheses and ambiguity. In addition, laboratories should not sequence data, re-analyze results, or report novel findings unless specifically requested to do so or for quality assurance purposes (88, 89).

### Genomic results reporting

Due to the importance of appropriately reporting the findings of genomic diagnostic tests, international guidelines exist and must be followed. The data should be presented in a manner that is easily understood by both patients and clinicians (90). The report should contain the reason the patient was referred for genetic testing and the genes tested based on the patient's phenotype. In the event of an NGS genetic test, the reasoning and databases used for gene selection must also be stated. The areas targeted by the assay must be explicitly characterized, and if only coding regions and flanking intronic sequences are included in the analysis, this must also be specified. Both the reference sequence used for alignment and the appropriate transcripts should also be included. In addition, an accurate description of the applied NGS technology should be provided, including details regarding the platform, the read depth, and the assay's sensitivity and specificity for identifying different types of variation (including CNVs) (88). Details about the bioinformatic techniques and tools used for variant calling and interpretation should also be supplied. P, LP, and VUS variants should be reported appropriately, and the suggested classification should be adequately supported using the classification criteria provided by the ACMG recommendations. Additional information should include the variant frequency in population databases and variant databases, as well as bibliographic publications detailing affected individuals who have the same variation. In addition, the defective gene(s) and their connection with the patient's phenotype should be described in detail (7). In the event that a VUS is reported, the ACMG criteria used for its categorization as well as any accessible in silico studies and the expected impact on the protein's function should be included. Also included should be a statement indicating that VUS should not be utilized for clinical decision-making (74). Due to the unnecessary confusion they create, it is not recommended to report B/LB variants.

### Genetic counseling

Genetic counseling from a competent genetic counselor should always be offered to the proband and family both before and after the genetic analysis (29, 44, 62, 91). Pre-test genetic counseling is of paramount relevance. The collecting of all clinical information about the proband and his or her family should be the first step in determining the possibility of a genetic etiology for the condition. Thus, a pedigree is generated using information from at least three generations about pathological conditions in the family (92). Moreover, a clear explanation of the purpose of such an analysis, as well as information regarding the genes being studied should be offered (44). Moreover, it is imperative to notify patients and/or their families regarding the likelihood of identifying variants that may predispose them to a disease beyond the primary clinical inquiry (referred to as unsolicited or incidental findings) in the context of performing a WES or WGS. In addition, they should be informed about the possibility to obtain results in genes defined as clinically important by international recommendations and to whom it is advised that the results are reported, regardless of the reason for referral. The current ACMG list for reporting secondary findings in clinical exome and genome sequencing consists of 73 genes (93). In addition, a clear picture of the potential outcomes of the analysis must be presented, informing about the possibility of rendering positive, and negative but also VUS results.

In the event of a negative result, it should be made clear, especially in the case of a family history of the disease, that the genetic test cannot rule out the presence of an inherited condition. Several factors may have contributed to the absence of a causal variation. It might be attributed to a lack of assessment of the affected gene due to missing information about the genes implicated in the disease or to the incapacity of the technology utilized to detect the causal variation (for example deep intronic variants or certain types of CNVs). In this instance, family members cannot be offered genetic predisposition testing to identify their risk (7). Therefore, all first-degree family members should continue to be regarded as being at risk for the disease and should continue to complete the suggested family monitoring. In the future, genetic testing may be reconsidered if new knowledge on the genetic origins of the ailment becomes available (7, 74).

When a VUS is identified, the same course of action should be taken as when a negative result with a positive family history is obtained. Informing the proband and family that the finding does not exclude the probability of an inherited cardiological disorder in the family is suggested. In anticipation of a potential future reclassification of VUS, the suggested surveillance of the proband and at-risk family members must be continued (74).

When the genetic test yields a positive result, the gene variant responsible for the disease has been identified. This discovery confirms the genetic etiology of the disease and has diagnostic ramifications. It contributes to the treatment of patients, family cascade testing, and, in certain instances, the direction of therapeutic selections. Upon completion of the test, the results of the genetic analysis should be clearly disclosed to the affected parties, and if required, they should be sent to additional medical specialties for treatment advice, monitoring, and psychological support (90).

In certain circumstances, the requested genetic investigation yields negative findings for a particular genetic ailment, but the geneticist may indicate the possibility of a different Mendelian disease throughout the genetic counseling process. Due to the introduction of comprehensive NGS genomic tests and the use of virtual panels, it is now possible to evaluate genes connected to a disease suspected by the physician, as well as scan other genes in the event of a negative test, therefore increasing the phenotypes analyzed (90).

## Polygenic risk score (PRS)

Predicting diseases or phenotypes using genetic risk based on Genome-wide association studies (GWAS) has become increasingly popular in recent years (94). Such an approach has added to the identification of new genes and genetic loci that contribute to an increased risk for several CVDs (95).

In addition, polygenic risk scores (PRS) have been developed by the integration of these findings. These scores evaluate an individual's genetic susceptibility to a trait or condition, based on their genotype profile and relevant (GWAS) data, by calculating the cumulative impact of low to intermediate-risk variants in a patient group (96–98). PRS is anticipated to be a prediction and risk stratification tool for identifying people with a greater tendency to complex CVD disorders, and it has the potential of shedding light on the molecular basis and the prediction of age-dependent clinical outcomes. For several CVDs such as Atrial Fibrillation, Coronary artery disease (CAD), and Type 2 Diabetes, the predictive accuracy of established clinical risk factor models is improved with the addition of PRS. PRS enhances prediction in several risk models such as the PCE risk model for Atherosclerotic cardiovascular disease (ASCVD), the CHARGE-AF risk model for Atrial Fibrillation, and the American Diabetes Association risk model for Type 2 Diabetes (96). PRS risk tools may identify individuals at risk for developing a disease, allowing preventative measures to be taken. For example, individuals having a high PRS for CAD get the most relative and absolute benefit from LDL cholesterol-lowering treatments, according to *post hoc* analysis of completed clinical studies. Moreover, PRS for CAD might be used to identify patients who would benefit from extensive lifestyle adjustment, imaging monitoring, and early statin medication (99).

The utility of PRS can also be expanded in cardiac disorders that are considered traditionally of monogenic etiology. Since most of these conditions remain genetically undiagnosed, the addition to PRS could add to the identification of individuals at risk without identified gene alteration in one of the causative genes. For example, it has been shown that PRS could shed light on the genetic origins of HCM beyond the Mendelian paradigm of single-gene inheritance (98, 100–102). Moreover, it could also explain the differential variant penetrance observed and the interindividual variability in disease severity among individuals with HCM or DCM carrying the same pathogenic variant (100, 102). Therefore, an increasing body of evidence points to clinically significant roles for genetic risk stratification in CVD. However, prior to being extensively adopted in clinical practice, more prospective studies examining the clinical value of PRS are needed (103).

## Clinical utility of genetic analysis

Genetic analysis confirms the clinical diagnosis reliably and facilitates quick diagnosis by evaluating all possible disease-causing genes simultaneously. Obtaining a conclusive molecular diagnosis permits the clinician to set up appropriate, potentially life-saving surveillance or referrals. In certain circumstances, a confirmed molecular diagnosis may lead to modified medical management and treatment. Based on the findings of the genetic analysis and the identified genetic variation, relevant lifestyle modification advice might be provided to lower the likelihood of a cardiac event or sudden cardiac death. It has been shown that the chance of developing ventricular arrhythmias rises with exercise. Patients with arrhythmogenic cardiomyopathy and LQT1 should thus be prevented from participating in competitive or endurance exercises. In addition, it might aid in deciding whether to install a cardiac defibrillator or pacemaker (104). In certain instances, enzyme replacement medication, early surgery, and heart transplantation are potentially viable treatment options (Table 5).

**Table 5:** List of genes and the associated diseases that could lead to modification of patients’ management.

Phenotype	GENE	Type of intervention	Therapeutic intervention	Strength of Evidence	References
DCM	*LMNA, RBM20, PLN and FLN*	Management modification/surveillance	Higher risk of SCD: early indication of ICD implantation should be considered	Guidelines	(105)
DCM	*LMNA*	pharmacotherapy	Lovastatin	Limited/Preclinical	(106)
ACM	*FLNC, DSP, LMNA, DES* and *PLN*	Intervention/surveillance	In ACM patients carrying truncating variants with reduced LV systolic function an ICD is reasonable	Guidelines	(7, 107)
Pompe disease (HCM phenocopy)	*GAA*	Treatment	Enzyme-replacement therapy, noninvasive ventilation	Strong/approved treatment	(63)
Anderson-Fabry disease (HCM phenocopy)	*GLA*	Treatment	Antiplatelet/anticoagulant agents, enzyme replacement therapy, analgesic drugs to relieve neuropathic pain	Strong/approved treatment	(63)
Danon disease (HCM phenocopy)	*LAMP2*	Intervention	ICD implantation	Strong/approved treatment	(63)
Wolff-Parkinson-White syndrome (HCM phenocopy)	*PRKAG2*	Treatment recommendations	Antiarrhythmic drugs, ablation	Limited/Single Study	(108)
Transthyretin amyloidosis (HCM phenocopy)	*TTR*	Intervention	Liver/Kidney/heart transplantation	Strong/approved treatment	(63)
LQT1	*KCNQ1*	Treatment recommendations	b-blockers, left stellectomy	Strong	(109)
LQT1	*KCNQ1*	Management recommendations	Avoid exercise (swimming)	Strong	(109)
LQT2	*KCNH2*	Treatment recommendations	b-blockers, left stellectomy, Potassium suppletion	Strong	(109)
LQT2	*KCNH2*	Management recommendations	Avoid arousal	Strong	(109)
LQT3	*SCN5A*	Treatment recommendations	b-blockers, Sodium channel blockers, pacemaker	Strong	(109)
LQT	*CACNA1C, CALM1, CALM2, CALM3, KCNE1, KCNH2, KCNJ2, KCNQ1, SNC5A, TRDN*	Management recommendations	ICD for symptomatic patients, in spite of beta-blocker therapy, to prevent SCD. Pacemaker for patients who have an abnormally slow heart rate. Avoid certain drugs which decrease repolarisation reserve or hypokalaemia	Strong	(109)
SQT	*KCNH2, KCNQ1, KCNJ2, SLC4A3, SLC22A5, CACNB2, CACNA2D1, CACNA1C, SCN5A*	Management recommendations	ICD Anti-arrhythmia drugs, such as quinidine.	Strong	(110)
Brugada	*SCN5A*	Management recommendations	If ventricular arrhythmias or an aborted SCD, consider ICD	Strong	(111, 112)
FH	*LDLR, APOB, PCSK9, LDLRAR1*	Treatment recommendations	intense Statin	Strong	(113, 114)
FH	*PCSK9, Heterozygous FH*	Treatment recommendations	PCSK9 inhibitor	Strong	(113, 114)
Non Syndromic HTAD	*ACTA2, MYH11, MYLK, PRKG1*	Surgical intervention	Dissection if aorta size 4.5–5cm	Strong	(115–118)
Non Syndromic HTAD	*MYLK*	Surgical intervention	Dissection may occur without dilatation	Supporting	(117)
Marfan syndrome	*FBN1*	Surgical intervention	Dissection if aorta size ≥4.5–5 cm depending on family history and other risk factors	Strong	(117, 119)
Marfan syndrome	*FBN1*	Treatment recommendations	Beta blockers	Supporting	(117)
Loeys-Dietz syndrome	*TGFBR1, TGFBR2, SMAD3*	Surgical intervention	Dissection if aorta size 4.0–4.5 cm	Strong	(117, 120)
Loeys-Dietz syndrome	*TGFB2*	Surgical intervention	Dissection if aorta size ≥4.5 cm	Supporting	(117, 120)
Loeys-Dietz syndrome	*SMAD2, TGFB3*	Surgical intervention	Dissection if aorta size ≥5 cm	Supporting	(117, 120, 121)
Vascular Ehlers-Danlos syndrome	*COL3A1*	Surgical intervention	Increased risk of complications from vas-cular surgery because of friable tissue. Multidisciplinary approach required	Strong	(117, 120, 122)

ICD, Implantable cardioverter-defibrillator; SCD, Sudden cardiac death; HTAD, Hereditary aortic disease; FH, Familial Hypercholesterolemia; LQT, Long QT; SQT, Short QT.

In dilated cardiomyopathy (DCM) for example, according to the guidelines, for patients with P/LP variants in *LMNA, RBM20, PLN* and *FLN* genes, early implantable cardioverter-defibrillator (ICD) implantation should be considered due to the higher risk of sudden cardiac death (105). Patients harboring specific variants in *SCN5A* gene respond well to treatment with sodium-channel blocking drugs, while conventional heart failure therapy seems to be relatively ineffective (31, 123). The prospect of pharmacologically treating people who have not yet exhibited symptoms but have relevant variants is an intriguing aspect of the care of patients with DCM (positive genotype-negative phenotype). In this context, two clinical studies have demonstrated that using carvedilol or eplerenone upfront may improve the result (124).

In HCM, the genetic analysis of HCM phenocopies and the identification of the gene implicated in the disease are essential for the management of the patient, as the treatment in each of these cases is unique and gene-specific (45, 63). HCM phenocopies resembling sarcomere-gene-associated HCM are considered several lysosomal storage diseases such as LAMP-2 (Danon) cardiomyopathy, PRKAG2 syndrome, Fabry disease, Noonan syndrome and other RASopathies as well as transthyretin cardiac amyloidosis (45).

In ARVC, which is the best characterized ACM, genotype–phenotype correlation studies showed that patients carrying specific pathogenic variants can benefit from ICD implantation as primary prevention. In order to prevent Sudden Cardiac death (SCD) and according to current guidelines, implantation of an ICD is reasonable for patients carrying pathogenic variants in one of these genes: Phospholamban, FLNC, or lamin A/C with LVEF <45%. This could be the case as well as for two other genes: BAG3 and TMEM43, because LV dysfunction is most often present in patients with ACM and pathogenic/likely pathogenic variants in *BAG3, TMEM43*, as in *PLN* and *LMNA* genes and in a large study with patients carrying p.S358l variant in TMEM43, survival was greater for those who received an ICD than for those they did not (31, 107).

In Long QT syndrome (LQTS), genotype-phenotype associations have also been identified, as swimming and exertion-induced cardiac events are more prevalent in LQT1, auditory triggers and postpartum period events are more prevalent in LQT2, and a sleep-related event relates to LQT3. Likewise, pharmaceutical therapy might vary (124). In addition to the QTc length, the LQT syndrome genotype independently influences the risk of life-threatening arrhythmic events. At any given QTc, the risk for such events is much greater for patients with LQTS2 and LQTS3 (130% and 157%, respectively) than for patients with LQTS1, whereas the estimated hazard rises by 15% with each 10-ms increase in QTc length for all three genotypes (125). The pharmaceutical treatment with beta-blockers is the cornerstone of LQTS therapy, reducing the necessity for defibrillator implantation (126). Beta-blockers may lower the incidence of cardiac events by about 95% in patients with LQTS1, 75% in patients with LQTS2, and 80% in patients with LQTS3. Addition of sodium-channel blockers such as mexiletine to beta-blockers may be advantageous for individuals with LQTS3, and perhaps other subtypes such as LQTS2 (125).

Genetic testing in patients with familial hypercholesterolemia (FH) can provide prognostic and risk stratification information. According to data from the Myocardial Infarction Genetics Consortium patients with both LDL cholesterol ≥190 mg/dl and an FH variant show a 22-fold increased risk for CAD compared with individuals with LDL cholesterol <130 mg/dl and no variant, whereas for patients with LDL cholesterol ≥190 mg/dl and no FH variant the risk for CAD is 6-fold higher, and the presence of an FH pathogenic variant increases CAD risk >3-fold at the same LDL-C level (6, 127). Genotype-phenotype correlations exist, and genotype can drive pharmacotherapy as well as its time of initiation. Null variants in the *LDLR* gene are more severe than *LDLR*-defective, *APOB,* and *PCSK9* pathogenic variants affecting the degree of hypercholesterolemia as well as CAD and premature CAD risk. Furthermore, the distinction of FH arising from deleterious monogenic variants in FH-causing genes from a polygenic cause, has implications for the long-term response to therapy and the risk of atherosclerosis. FH due to a monogenic origin is linked with decreased response to traditional cholesterol-lowering therapy, as well as an increased burden of coronary atherosclerosis and risk of atherosclerosis-related events (97, 113). Thus, important prognostic information may be gleaned from genetic testing for hypercholesterolemia.

Although FH can be diagnosed clinically, genetic testing is required to confirm the diagnosis. Moreover, positive FH genetic testing can have an impact on the initiation of lipid-lowering therapy (LLT), adherence to therapy and LDL-C reduction, even in patients already receiving LLT, since detection of a FH pathogenic variant indicates higher CAD risk and the need for more aggressive LDL-C reduction. The significance of genetic testing for LLT is great since patients with severe Heterozygous Familial Hypercholesterolemia (HeFH) or Homozygous Familial Hypercholesterolemia (HoFH) can benefit from specific therapeutic choices. Lomitapide and mipomersen are approved only for HoFH. Although PCSK9 inhibitors are approved, among other indications, for clinically diagnosed FH patients, patients with gain-of-function *PCSK9* variants are remarkably responsive to this therapy, while in patients with HoFH and 2 LDLR null alleles), PCSK9 inhibition has no effect on LDL-C level, but if at least one allele had residual LDLR activity, PCSK9 inhibitors can lower LDL-C levels by approximately 35%. Apart from adult patients, genetic testing is beneficial for children with FH as well. In HeFH pediatric patients, statin treatment should be initiated from as early as 8–10 years of age and interventions promoting a healthy lifestyle should begin earlier. For children with HoFH, aggressive treatment is required at the time of diagnosis (91).

Heritable thoracic aortic diseases (HTAD) have a definite genetic basis with 20% of the patients having a positive family history and a significant mortality rate if untreated (128). They may be syndromic (related to concomitant clinical manifestations) such as Marfan and Loeys–Dietz syndromes or non-syndromic (as with *ACTA2, MYLK,* and *MYH11* gene variants). Early diagnosis, prompt monitoring, preventative treatment, and family screening are essential for addressing these conditions. Genetic testing could assist in accurate diagnosis and prognosis, as well as in guiding patients' management (115, 121, 122). The identification of the causative gene could provide information about the disease progression severity. Moreover, genetic findings may be used to determine the most effective surgical intervention strategies for these individuals. Patients with HTAD-associated variants that place them at high risk for dissection could be identified through genetic screening testing (116).

Results of genetic analyses may offer useful information not only to individuals with cardiac conditions but also to relatives at increased risk of developing the same disorder. Thus, cascade analysis of at-risk relatives for the variant detected in the proband should be offered and could lead to proper surveillance and management in case of a positive result. While unnecessary anxiety might be avoided if the relative examined does not carry the proband's pathogenic variant.

Moreover, genetic testing is imperative in individuals who suffered unexplained cardiac arrest or SCD as a high proportion of these incidents are due to inherited cardiac diseases (129). A disease-causing variant is identified in 10% of undiagnosed cardiac arrest survivors and in 18%–60% of SCD victims (130, 131). It is well known that SCD may be the first manifestation in some cases of inherited arrhythmia syndromes and cardiomyopathies, with the cause not being identified until the postmortem genetic investigation is performed (132). It has been shown that the incorporation of genetic testing into the autopsy investigation significantly enhances the identification of a potential cause of SCD in young individuals (133, 134). Therefore, the guidelines advocate conducting a postmortem genetic test on the SCD victim with the aim to diagnose inherited cardiac diseases, followed by a phenotype-based evaluation of the victim's relatives (129). In the event that a pathogenic variant is identified, the implications of such a strategy could be lifesaving for the family members, leading to appropriate surveillance and management of the relatives at risk.

Moreover, using preimplantation genetic diagnosis (PGD) or non-invasive prenatal testing, genetic analysis might be utilized to prevent the transmission of a potentially lethal gene alterations to future generations (135). Carriers with dominant pathogenic variants or partners both harboring recessive pathogenic variations may benefit from *in vitro* fertilization with PGD for the implantation of embryos unaffected by a life-threatening variant (62).

### Future prospective of CVDs therapies

Current therapies for CVDs consist mostly of conventional pharmacotherapy or interventional and surgical procedures (136–138). Although both relieve the symptoms associated with the disease and improve prognosis, they have some disadvantages. Traditional treatment may negatively affect the liver, kidneys, and other organs, while the practical application of cardiac surgery is perpetually constrained by the complexity of the operations and the risk of postoperative complications (139, 140). Therefore, novel therapeutic options with greater effectiveness and fewer side effects are necessary.

In the era of targeted therapy, proper disease management necessitates the utilization of biomarkers that could inform prognosis, diagnosis, treatment monitoring, along with treatment selection. Especially for treatment selection, it is imperative to utilize appropriate predictive biomarkers (141). However, in cardiology, the term “predictive biomarker” is not well established, and in some instances, it is improperly used to designate biomarkers that foretell the onset or progression of the disease without regard to treatment (142). Nevertheless, by definition, a predictive biomarker should be used to predict a disease's progression in correlation to a specific treatment selection (Such biomarkers are successfully employed in the fields of oncology and hematology, where hereditary or somatic genetic abnormalities are targeted by specialized treatment regimens and are therefore used as predictive biomarkers to identify patients suitable to undergo such targeted treatments (143–146).

Compared to nonselective therapeutic treatments, gene-directed treatment techniques have demonstrated superior clinical efficacy for a number of cancers. Non-small cell lung cancer is an example of the tumor type with the greatest number of available biomarkers and targeted treatments. Currently, somatic gene variant analysis is required for defining the appropriate first or subsequent targeted therapy lines, whereas medical recommendations encourage determining treatment decisions based on genomics data (147–149). Similarly, in ovarian breast and pancreatic cancers, variants in Breast Cancer genes 1 and 2 (BRCA1/2) are utilized as predictive biomarkers to identify patients who are most likely to react to Poly (ADP-ribose) polymerase (PARP) inhibitors (150).

Since the genomic analysis is increasingly applied to more patients, it is becoming evident that several gene alterations could be appropriate biomarkers for identifying patients eligible for targeted treatments in various medical specialties, including cardiology. Conversely, individuals with different genetic causes of heart failure, such as hypertrophic cardiomyopathy (HCM), cardiac amyloidosis, or Fabry disease, already have access to a variety of disease medications (151, 152). In addition, two monoclonal antibodies evolocumab and alirocumab have been approved approved by the FDA in 2015 as an add-on to the therapy for heterozygous familial hypercholesterolemia (HeFH) and homozygous familial hypercholesterolemia (HoFH) (153).

Furthermore, a number of ongoing clinical studies seek to broaden the use of gene-informed therapy selection, and a number of targeted therapies with associated predictive biomarkers are anticipated to emerge in the near future (Table 6). Currently, protein drugs, gene editing technologies, nucleic acid medicines, and cell therapy are the most often investigated targeted therapies for cardiovascular disorders (CVDs).

**Table 6:** Gene informed clinical trials in cardiology (accessed on 06/12/2022).

Clinical Trial NTC number	Gene	Conditions	Interventions	Therapy mechanism of action	Phase	Location
**Cardiomyopathy (CM)**						
NCT03439514	*LMNA*	DCM with Lamin A/C Gene Variant	ARRY-371797	p38a (MAPK14)-selective kinase inhibitor	3	United States
NCT05321875	DCM genes	DCM	Drug: Candesartan	Angiotensin II type 1 receptor blocker	3	
NCT04519749	*GLA*	Fabry Disease	Biological: 4D-310	AAV gene therapy delivering a GLA transgene	1/2	United States
NCT04935021	*TTR*	Transthyroxine Amyloidosis CM	Drug: ATTR-CM	Recombinant AAV2/6 vector encoding the cDNA for human a-Gal A	4	China
NCT04601051	*ATTR*	Transthyretin-Related (ATTR) Familial Amyloid Cardiomyopathy	Biological: NTLA-2001	CRISPR therapy	1	New Zeeland, Sweden, United Kingdom
NCT05445323	*FXN*	Friedreich Ataxia|CM Secondary	Genetic: LX2006	AAV gene therapy delivering the human frataxin (hFXN) gene to cardiac cells	1/2	United States
NCT04046224	*GLA*	Fabry Disease	Biological: ST-920	Recombinant AAV2/6 vector encoding the cDNA for human a-Gal A.	1/2	United States
NCT05302271	*FXN*	Friedreich Ataxia|CM	Biological: AAVrh.10hFXN|Drug: Prednisone	Serotype rh.10 AAV gene therapy delivering the hFXN gene to cardiac cells	1	
NCT04040049	*GLA*	Fabry Disease|Lysosomal Storage Diseases	Genetic: FLT190	AAV delivering the wild type GLA gene	1/2	United Kingdom
NCT03882437	*LAMP2*	Danon Disease	Biological: RP-A501	Recombinant AAV serotype 9 containing the human LAMP2B transgene	1	United States
NCT04455230	*GLA*	Fabry Disease|Lysosomal Storage Diseases	Genetic: FLT190	AAV vector (AAVS3) containing the human *α*GLA gene as a ssDNA	Not applicable	United Kingdom
**Arrhythmias**						
NCT04581408	*KCNH2*	Long QT Syndrome	Drug: Lumacaftor/Ivacaftor, Orkambi Oral Tablet	Two small molecule therapies targeting defects of mutant CFTR channels	2	Italy
NCT05223725	*KCNH2-G628S*	Atrial Fibrillation	Biological: AdKCNH2-G628S	AAV gene transfer using the KNCH2-G628S gene variant, to prolong atrial action potential	1	
NCT05122975	*RYR2*	CPVT	Lumacaftor/Ivacaftor, Orkambi® Oral Tablet	RyR calcium release channel stabilizer	2	2
Familial Hypercholesterolemia						
NCT04948008	FH genes in homozygosity	FH	IBI306	Anti-PCSK-9 monoclonal antibody	2/3	China
NCT05398029	FH genes in heterozygosity	Heterozygous Familial Hypercholesterolemia	Drug: VERVE-101	Base-editing technology designed to disrupt the PCSK9 gene in the liver	1	New Zealand
NCT04659863	FH genes in homozygosity	HoFH	Drug: Inclisiran	PCSK9-interfering mRNA	3	United States
NCT04659863	FH genes in homozygosity	HoFH	Drug: Inclisiran	PCSK9-interfering mRNA	3	United States
NCT04031742	FH genes in homozygosity	HoFH	Biological: IBI306	Anti-PCSK-9 monoclonal antibody	2/3	China
NCT05217667	FH genes in homozygosity	HoFH	Drug: ARO-ANG 3 Injection	ANGPTL3 protein expression inhibitor; RNA interference	2	United States, Australia, Canada
NCT05325203	FH genes in heterozygosity	HeFH	Biological: Ongericimab|Drug: Placebo	Human anti-PCSK9 monoclonal antibody	3	China
NCT04681170	FH genes in homozygosity	HoFH	Drug: Lomitapide	Microsomal triglyceride transfer protein inhibitor	3	Germany, Israel, Italy, Spain,Tunisia
NCT05398029	*LDLR* in heterozygosis	HeFH	Drug: VERVE-101	Adenine base editing to knock out the PCSK9 gene	1	New Zealand
NCT05043181	FH genes in homozygosity	HoFH	Low Density Lipoprotein Receptor mRNA Exosomes	Low Density Lipoprotein Receptor mRNA Exosomes	1	China

Gene editing technology has the potential to cure many forms of inherited cardiovascular disease in the future. Theoretically, monogenic cardiovascular diseases could be eliminated from future generations of affected families through germline genome editing and repair. Even though this approach is theoretically feasible and will likely be available for human use in the near future, it is ethically questionable. Somatic genome editing may also be effective for a range of cardiovascular diseases, despite the fact that it is currently plagued by various technological obstacles and has not yet progressed beyond small animal models. It has the potential to cure individuals who are currently afflicted with diseases while avoiding ethical concerns regarding persistent germline modification (154).

The first generation of tools referred to collectively as engineered nucleases possess the capacity to precisely identify and bind to a particular genomic sequence, thereby inducing a double-strand DNA break within that sequence. Recent CRISPR-Cas9 gene editing technologies permit binding to the target sequence and have the capacity to modify it. The most prevalent techniques involve chemically modifying DNA bases (base editing), altering gene expression (epigenome editing), and using reverse transcription to incorporate new DNA sequences derived from RNA templates (prime editing) (154).

Base editing (BE) or prime editing (PE) was recently used to correct pathogenic variants in *MYH7* and *RBM20* induced pluripotent stem cell-derived cardiomyocytes (iPSC-CM) and humanized mouse models (155, 156). CRISPR-Cas9 has also been tested for the treatment of various cardiac disorders including cardiomyopathies (targeting genes such as *MYBC3* and *PLN*) (157, 158), Transthyretin cardiac amyloidosis (159), Inherited arrhythmic disorders (160, 161) and dyslipidemia and atherosclerotic cardiovascular disease (162), among others.

## Conclusions

Numerous medical disciplines, including cardiology, have benefited from the development of reliable and comprehensive genetic analysis due to technological advancements. Importantly, NGS-based genomic analysis led to an increase in the diagnostic yield compared to single gene analysis strategies, clarifying a diagnostically ambiguous picture, and delivering a precise and succinct diagnosis that is essential for proper patient management. The applicability and usability of the new NGS sequencing technologies are also contingent on the multidisciplinary teamwork required to guide each patient toward the genetic test that is most relevant based on his phenotype, using the most cost-effective technology. The expanded use of modern NGS technology is guiding future gene therapy clinical trials and increasing our understanding of the genetic component of several cardiac defects. Thus, it is anticipated that precision medicine and the use of gene-informed targeted therapies would soon become a reality. Prerequisites for the implementation of comprehensive NGS approaches in clinical practice though, is the appropriate analysis and interpretation of the complex results obtained from such analysis, which requires the implementation of advanced analytical procedures and resources. In this respect, a better comprehension of the genes and genetic variants involved in cardiac disorders is of paramount importance and is achieved primarily due to the accumulation of data generated from the rising number of analyses performed, with the assistance of international alliances and data-sharing initiatives.
